# Telitacicept following plasma exchange in the treatment of subjects with recurrent neuromyelitis optica spectrum disorders: A single‐center, single‐arm, open‐label study

**DOI:** 10.1111/cns.13904

**Published:** 2022-07-18

**Authors:** Jie Ding, Xianguo Jiang, Yu Cai, Shuting Pan, Ye Deng, Meichun Gao, Yan Lin, Nan Zhao, Ze Wang, Haojun Yu, Huiying Qiu, Yuyan Jin, Jiahui Xue, Quan Guo, Liping Ni, Ying Zhang, Yong Hao, Yangtai Guan

**Affiliations:** ^1^ Department of Neurology, Renji Hospital, School of Medicine Shanghai Jiaotong University Shanghai China; ^2^ Clinical Research Center, Renji Hospital, School of Medicine Shanghai Jiaotong University Shanghai China

**Keywords:** clinical study, effectiveness, neuromyelitis optica spectrum disorders, plasma exchange, safety, Telitacicept

## Abstract

**Introduction:**

Neuromyelitis optica spectrum disorders (NMOSD), mainly mediated by B cells and AQP4 antibody, has a high rate of recurrence. Telitacicept is a novel drug specifically targeting the upstream signaling for the activation of B cell with its following production of autoimmune antibodies. Thus, it may be a promising approach. Our study preliminarily explored the potential safety and effectiveness of Telitacicept following plasma exchange in the treatment of recurrent NMOSD.

**Methods:**

This was a single‐center, single‐arm, open‐label study enrolling eight patients with recurrent NMOSD in China. All patients received plasma exchange three times, followed by Telitacicept 240 mg every week for 46 times. The primary endpoint was the time of first recurrence after enrollment. Secondary end points included: changes in Expanded Disability Status Scale score, Optic Spinal Impairment Scale score, Hauser Ambulation Index, number of lesions on MRI, retinal nerve fiber layer thickness measured by optical coherence tomography, latency and amplitude of visual evoked potential, titer of AQP4 antibody, and immune parameters of blood. Safety was also assessed. The study was registered with Chictr.org.cn (ChiCTR1800019427).

**Results:**

Eight eligible patients were enrolled. Relapse occurred in two patients (25%) and five patients (63%) remained relapse free after 48 weeks of treatment. The time to first recurrence was prolonged and the number of recurrences was reduced (*p* < 0.001, power of test = 1). One patient withdrew from the study due to low neutrophil count. No serious adverse events occurred.

**Conclusions:**

In this small, uncontrolled study, Telitacicept following plasma exchange has the potential to be a safe treatment for patients with recurrent NMOSD. It may prolong the recurrence interval and reduces the annual count of recurrences. A multicenter randomized controlled study with a larger sample is thus feasible and needed to further assess its safety and efficacy.

## Graphical abstract

In this small‐scale study, Telitacicept following plasma exchange has the potential to be a safe and effective treatment for patients with recurrent neuromyelitis optica spectrum disorders (NMOSD). It may prolong the recurrence interval and reduces the annual count of recurrences. Although this work does not draw a definite conclusion concerning the safety and effectiveness, it provides important information of a novel alternative therapeutic strategy for NMOSD with probably better therapeutic potentials but with no safety issues observed so far, worthy of next stage clinical study.

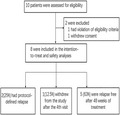

## 1 INTRODUCTION

Neuromyelitis optica spectrum disorders (NMOSD) is an autoimmune disease of the central nervous system (CNS) mainly mediated by B cells and AQP4 antibody.^1^ The prevalence rate is ∼0.5–4 per 100,000 and it can reach 10 per 100,000 in some ethnic groups.^2^ It has a high rate of recurrence and disability.^3^, ^4^ The reported incidence of NMOSD in female is up to 10 times higher than in men. The median age of onset is around 40 years old, with a high incidence in young and middle‐aged people.^5^, ^6^ NMOSD greatly impacts both individuals and society. Therefore, a safe and effective treatment targeting the pathogenesis of NMOSD is urgently needed.

Nowadays, the treatment of NMOSD varies according to the stage of the disease. In the acute stage of relapse, initial treatments involve high‐dose intravenous methylprednisolone, intravenous immunoglobulin^7^ and plasma exchange.^8^, ^9^ Plasma exchange has been proved to be effective in the treatment of acute‐phase NMOSD.^9^, ^10^, ^11^ It substantially filters the major pathogenic molecules, such as AQP4 antibody. In the remission phase, concomitant immunotherapy such as azathioprine and mycophenolate mofetil are used to prevent recurrence. Although there are some recent drug developments in phase III clinical trials in NMOSD,^12^ current immunosuppressant medications are used off‐label for the prevention and treatment in patients with NMOSD.^13^ Stepwise deterioration due to recurrent attacks and accumulated disability remain an intractable problem in patients with NMOSD. The side effects of glucocorticoid and current immunotherapy, such as leukocytopenia and osteonecrosis of femoral head, lead to poor compliance and outcome.^14^ Nowadays, clinical research in NMOSD is focusing on the development of novel medications and therapeutic strategies.^15^

Telitacicept (previously known as Atacicept or TACI‐Ig) is a recombinant human B lymphocyte stimulator receptor: IgG Fc fusion protein, targeting both B cells and T cells. The soluble part of transmembrane activator and calcium modulator and cyclophilin ligand interactor (TACI) in Telitacicept recognizes two key regulators of lymphocyte development and maturation: B‐lymphocyte stimulating (BLyS) and proliferation‐inducing ligand (APRIL), Therefore, Telitacicept effectively neutralizes the interaction among BLyS, APRIL and their receptors. TACI receptor is located on CD27+ Memory B cells and plasma cells. Telitacicept further hinders the proliferation of B lymphocytes and the maturation of T lymphocytes, preventing the occurrence of autoimmunity.^16^ The efficacy and safety of Telitacicept has been demonstrated in Phase IIb clinical studies in patients with systemic lupus erythematosus (SLE) or rheumatoid arthritis (RA).^17^, ^18^ Telitacicept has received its first approval for patients with SLE in March 2021 in China. Clinical studies of telitacicept are also undergoing in several other indications such as MS (NCT04625153), myasthenia gravis (NCT04302103), IgA nephropathy (NCT04291781 and NCT04905212) and Sjögren's syndrome (NCT04078386).^19^ Telitacicept was reported to be safe and effective in experimental autoimmune encephalomyelitis (EAE), an animal model of inflammatory demyelinating disease of the CNS.^20^ BLyS and APRIL are members of Tumor Necrosis Factor (TNF) family, predominantly found in B cell‐mediated autoimmune diseases such as SLE, RA and NMOSD rather than MS.^21^, ^22^, ^23^, ^24^, ^25^ Thus, it is plausible that Telitacicept may be a promising drug regimen in NMOSD.

Telitacicept is a novel drug specifically targeting the upstream signaling for the activation of B cell with its following production of autoimmune antibodies. It is thus plausible to speculate upon the therapeutic potential of Telitacicept in prolonging the effectiveness of plasma exchange, which eliminate the existing autoimmune antibodies in the circulation. According to our knowledge, we are the first group to investigate the outcome in the treatment of recurrent NMOSD with Telitacicept following plasma exchange. As the strict regulations and high‐standard requirements for initiating clinical research in Shanghai, we are only allowed to conduct a single‐center clinical study of Telitacicept in a small scale due to the registered indications of Telitacicept is limited to SLE only. Therefore, the objective of this study is to investigate preliminarily, for the first time, the potential safety and efficacy of Telitacicept following plasma exchange in treating recurrent NMOSD, providing insight for future large‐scale multicenter randomized controlled trials.

## 2 METHODS

### 2.1 Study design

This was a single‐center, single‐arm, open‐label study. It included eight patients with recurrent NMOSD admitted to the Department of Neurology, Renji Hospital Affiliated to Shanghai Jiao Tong University School of Medicine, from June 2019 to October 2020.

### 2.2 Participants

Patients were diagnosed as NMOSD according to the criteria defined by the 2015 international consensus diagnostic criteria of NMOSD.^1^ Acute phase is defined as new onset of neurological symptoms or aggravation of existing symptoms within 30 days before screening, lasting for at least 24 h without accompanying fever. The inclusion criteria were (1) NMOSD in acute phase, (2) had no less than two episodes of recurrences (including the acute recurrence at screening) for the last 12 months before the screening, (3) aged from 18 to 75 years old.

Patients meeting the following conditions were excluded from the study. (1) progressive nervous system symptoms not related to NMOSD; (2) active hepatitis or history of severe liver disease (more than twice the normal value of liver function test). Patients with positive HBsAg surface antigen were not eligible for enrollment. But patients with single positive anti‐HBC needed to be tested for the quantity of HBV‐DNA. If the quantitative detection of HBV‐DNA was negative, they could be considered eligible for the study. (3) Patients with renal insufficiency (including acute kidney injury or chronic kidney disease with an estimated serum creatinine clearance rate <60 ml/min calculated according to Cockcroft Gault equation); (4) pregnant women, lactating women or patients planning to conceive in the next 48 weeks; (5) patients who received any clinical trial drug within 28 days before enrollment or within 5‐fold half‐life of the trial drug (taking the shorter time); (6) patients who underwent splenectomy; (7) patients with a positive history for reaction to contrast agents for parenteral route of administration or human derived biological products; (8) patients with severe mental illness symptoms, who were unable to collaborate; (9) patients unable to undergo MRI examination; (10) patients who had used rituximab or mitoxantrone during 3 months before enrollment.

### 2.3 Procedures

Specific details regarding this study procedures, can be found in our study protocol published previously.^26^ All patients received plasma exchange three times. Telitacicept was then administered subcutaneously 14 days after enrollment, with a dose of 240 mg once a week (at least 48 h apart) for 46 times in total. After registration, participants received no systemic corticosteroids or immunosuppressive agents such as azathioprine, mycophenolate mofetil, cyclosporine and methotrexate. Biological immunosuppressive agents, hematopoietic stem cell transplantation, lymphatic irradiation and immunoglobulin injection were also prohibited.

During the study, visits were conducted at 4th, 12th, 24th and 48th week after enrollment. Expanded Disability Status Scale (EDSS) score, Optic Spinal Impairment Scale (OSIS) score and Hauser Ambulation Index were evaluated by two senior neurologists. Patients who had a protocol‐defined recurrence were recorded and withdrawn from the study. A recurrence was defined by the following criteria: (1) the appearance of new nervous system abnormalities or deterioration of the original symptoms; (2) the symptoms last for at least 24 h; (3) more than 30 days from the last recurrence; (4) without fever, body temperature <37.5°C without infection.^27^

Blood and urine tests were performed at the baseline, 4th, 12th, 24th and 48th week after enrollment or recurrence, including virological examination, pregnancy test, complete blood count, serum biochemicals, immunoglobulin, complement, lymphocyte subsets count and urine routine. Optical coherence tomography (OCT) was used to evaluate the changes in retinal nerve fiber layer (RNFL) thickness according to “Fast RNFL Thickness” protocol.

Visual evoked potential (VEP) was used to record the latency and amplitude of P100. Titers of AQP4 antibody, BLyS and APRIL were examined at baseline, 24th and 48th week after enrollment. The titer of AQP4 antibody was detected by cell‐based transfection immunofluorescence assay (CBA), BLyS and April by enzyme‐linked immunosorbent assay (ELISA). Brain, optic nerve and spinal cord MRIs were performed at baseline, 48th week or in case of recurrence. The number of low‐signal lesions on T1 weighted images (T1WI), high‐signal intensities on T2 weighted images (T2WI) and gadolinium enhanced T1WI, were recorded by 3T MRI. The MRI report was interpreted by the radiologists of Renji Hospital Affiliated to Shanghai Jiaotong University School of Medicine.

Safety laboratory tests performed at screening, 4th, 12th, 24th and 48th week after enrollment or recurrence, including virological examination, complete blood count, serum biochemicals, urine routine and electrocardiography. Computed tomography (X‐ray) scans of chest performed at screening, 24th and 48th week after enrollment. Additional safety evaluations included physical examinations, assessment of adverse events (AEs) at each study visit. In study protocol published previously, cut‐off values were indicated when to withdraw or reduce the dose of Telitacicept.^26^

### 2.4 Outcomes

The primary endpoint of this study was the time of first recurrence after enrollment. Secondary end points included: changes in number of attacks in the year before and after enrollment, changes in EDSS score, OSIS score, Hauser Ambulation Index, number of lesions on MRI, RNFL thickness by OCT, latency and amplitude of VEP, titer of AQP4 antibody, IgG, IgM, IgA, complement C3, C4, absolute value of lymphocyte subsets, BLyS, APRIL and other blood immune parameters. Adverse events were also recorded for safety assessment. The evaluation was based on common terminology criteria for adverse events (CTCAE) 4.03.

### 2.5 Statistical analysis

Baseline characteristics were summarized by proper statistics according to the type of each variable. For efficacy analysis, numbers of each patient's attacks before enrollment, in the year before enrollment, and in the year after treatment were presented. Time until first attack after enrollment, which is the primary endpoint, was summarized in days, and its survival curve was generator by Kaplan–Meier estimator. Median recurrent interval before treatment was also calculated for each patient. For secondary efficacy outcomes, we conducted Wilcoxon rank test to test the changes in number of attacks in the year before and after enrollment, and used median (range) or frequencies (%) for summarizing other quantitative outcomes listed in Outcomes. For safety analysis, number of patients with AEs and number of events were both presented. All tests were two sided, and *p* values of less than 0.05 were considered as significant. All analyses were conducted using R (version 4.0.3). No imputation was applied for missing data.

### 2.6 Standard protocol approvals, registrations, and patient consents

The study has been approved by the Ethics committee of Renji Hospital Affiliated to Shanghai Jiao Tong University School of Medicine (No 2018‐088). Written informed consent was obtained from all participants. The protocol was registered with Chictr.org.cn (ChiCTR1800019427).

## 3 RESULTS

### 3.1 Baseline characteristics

**FIGURE 1:**
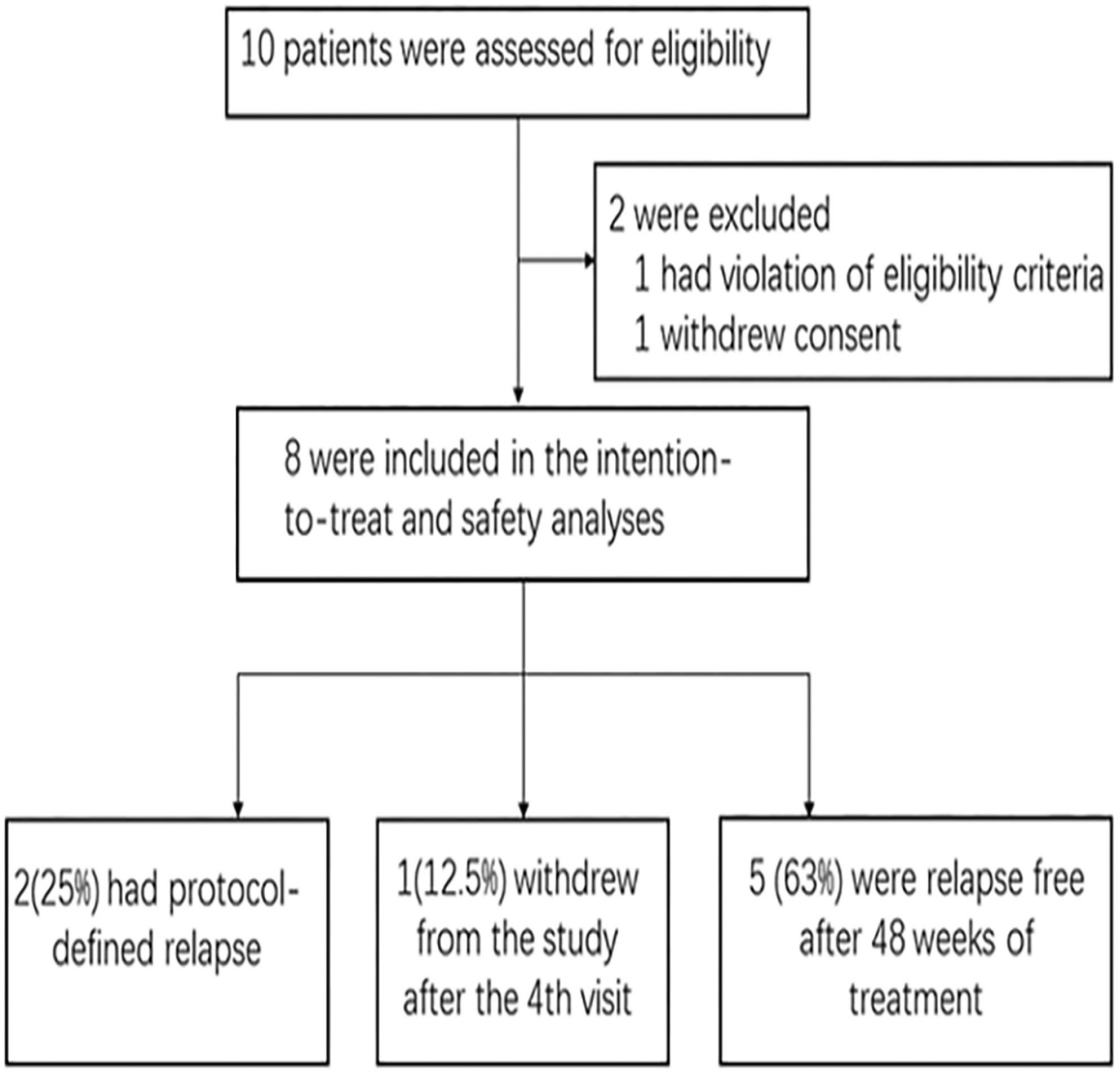
Enrollment and follow up of the participants

A total of ten patients were screened, of which eight met the inclusion criteria and were enrolled in the study (Figure 1). One patient was eliminated because of withdrawal of informed consent and the other one met the exclusion criteria of history of severe liver disease (more than twice the normal value of liver function test). Among eight patients, females accounted for 87.5% (*n* = 7) and males for 12.5% (*n* = 1). The age of the enrolled subjects ranged from 24 to 66 years with a median age of 53 years. All patients are seropositive to anti‐AQP4. The patient characteristics are listed in Table 1. Three patients failed to receive or tolerate attack prevention therapies before enrollment. Five patients received immunosuppressant treatments before enrollment. Regardless of prior drug regime, the recurrence rate remains high in all eight patients. The times of relapses prior to registration was 3 (2‐7) and the duration of disease was 1.20 (0.14, 18.43) years (Table 2).

**TABLE 1:** Baseline characteristics of participants

Index	Patients enrolled (*n* = 8)
Age at enrollment (years), median (range)	53 (24–66)
Sex, *n* (%)	
Male	1 (12.5)
Female	7 (87.5)
Ethic origin, *n* (%)	
Asian	8 (100)
Profession, *n* (%)	
Manual labor	2 (25.0)
Non‐manual labor	6 (75.0)
History of childbearing, *n* (%)	5 (71.4) (*n* = 7)
Times of pregnancy	2 (1–2) (*n* = 5)
Times of abortion	0 (0–1) (*n* = 5)
Smoking, *n* (%)	1 (12.5)
Comorbidity, *n* (%)	
Hypertension	3 (37.5)
Diabetes	1 (12.5)
Gallbladder stones	1 (12.5)
Systemic lupus erythematosus	2 (25.0)
Rheumatoid arthritis	1 (12.5)
Chronic gastritis	1 (12.5)
Hashimoto's thyroiditis	1 (12.5)
Duration of disease (years), median (range)	1.20 (0.14, 18.43)
Total number of previous attacks at enrollment	33
Number of previous attacks per patient, median (range)	3 (2–7)
Expanded Disability Status Scale (EDSS), median (range)	3.5 (2.5–4.5)
Optic spinal Impairment Score (OSIS), median (range)	
Visual acuity	6.0 (2.0–8.0)
Motor	1.0 (0.0–3.0)
Sensory	2.0 (0.0–3.0)
Sphincter	0.0 (0.0–1.0)
Hauser Ambulation Index, median (range)	1.0 (0.0–3.0)

**TABLE 2:** Attack prevention treatments and attack history before and after enrollment

Index	Attack prevention treatments before enrollment	Attacks before enrollment	Attacks in the year before enrollment	Attacks in the year after enrollment	Recurrent Intervals before treatment, days (median)	Time until first attack after enrollment, days
Patient 1	Unable to tolerate AZA and PDN	3	2	0	205.5	
Patient 2	Naïve	2	2	0	60.0	
Patient 3	Failed MMF and PDN	7	2	0	95.0	
Patient 4	Naïve	2	2	1	31.0	234
Patient 5	Failed AZA	6	2	0	1461.0	
Patient 7	AZA and tapering PDN for <45 days	3	2	0	61.5	
Patient 9	Failed AZA	3	2	1	192.5	45
Patient 10	Failed AZA and PDN	7	3	0	225.0	
*p*‐value				<0.001^a^		

Abbreviations: AZA, azathioprine; MMF, mycophenolate mofetil; PDN, prednisone.

^a^ Variance of number of attacks before and after enrollment.

### 3.2 Recurrent status of patients

**FIGURE 2:**
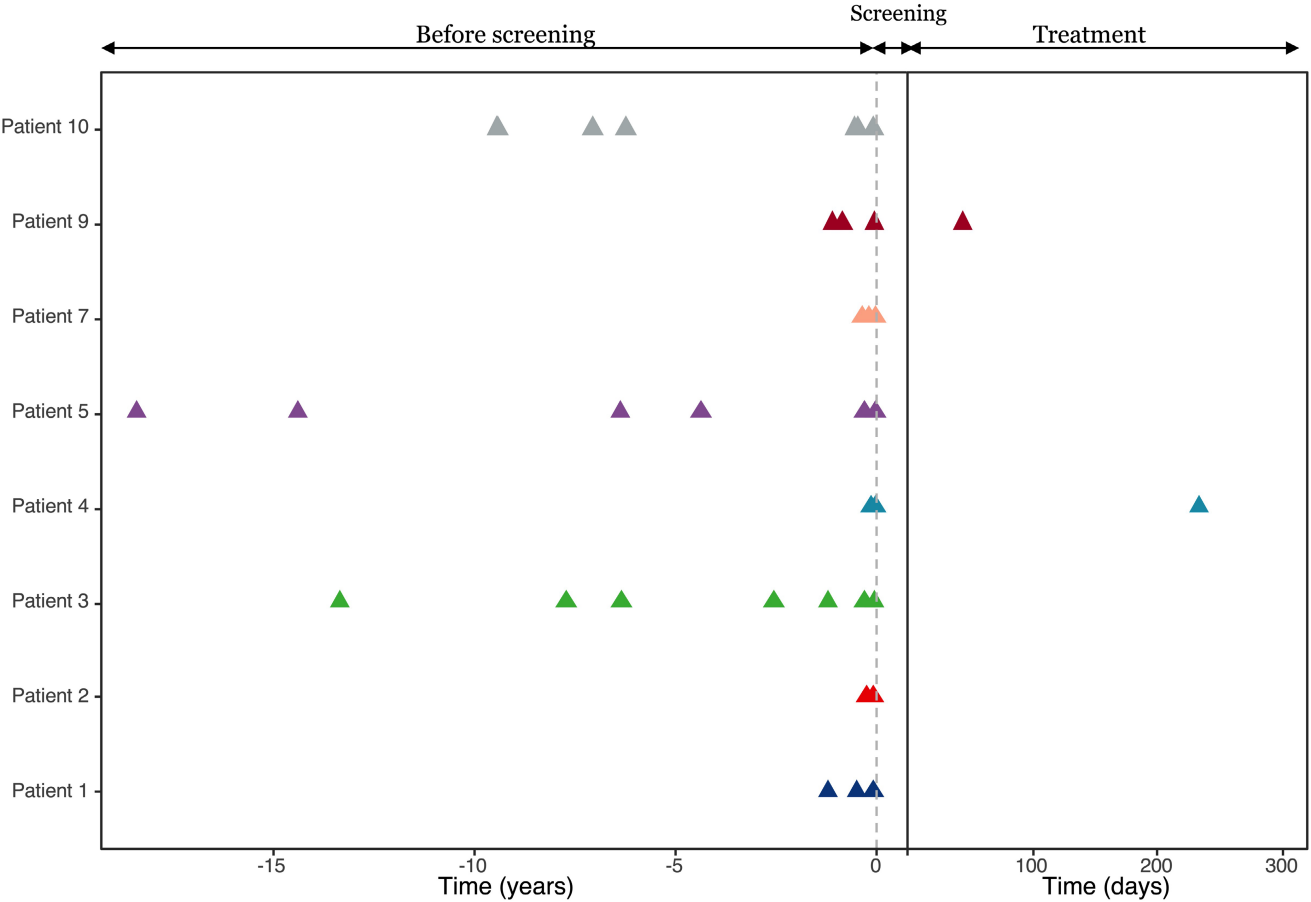
Attack frequency before and during Telitacicept treatment

**FIGURE 3:**
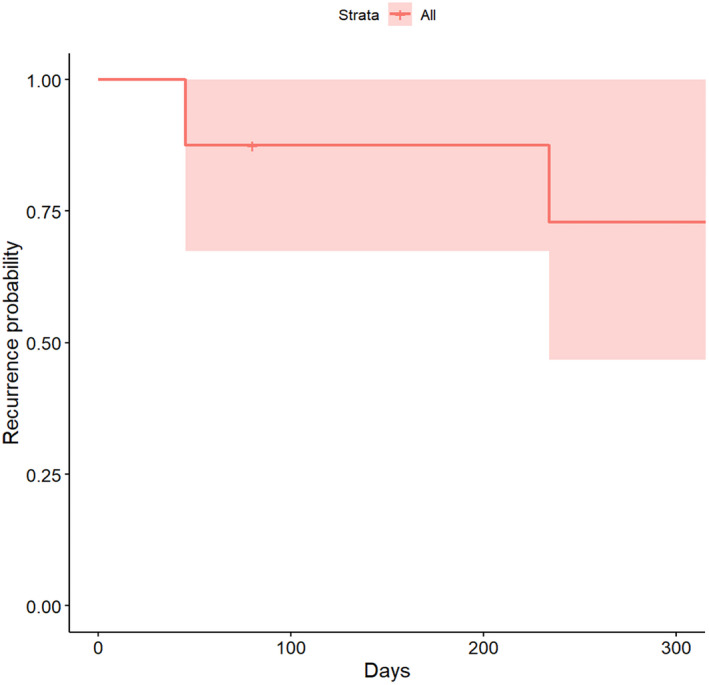
Recurrence probability during Telitacicept treatment

Five patients had no attack during the 48 weeks of treatment. One patient withdrew from the study due to low cell count of leukocyte and neutrophil. The administration of Telitacicept was ceased in the remaining two patients due to a single episode of NMOSD relapse and intravenous dose of methylprednisolone and azathioprine was given. Patient 4 relapsed 234 days after enrollment and Patient 9 relapsed on day 45. Compared with the year prior to registration, the time of first recurrence after enrollment was longer than previous recurrent intervals, and the annual number of attacks was reduced since enrollment (*p* < 0.001, power of test = 1; Table 2; Figures 2 and 3). Two patients with relapse both had preceding fatigue followed by optic neuritis sparing spinal cord. The EDSS score increased by 2 points.

After 48‐week treatment, EDSS score was ameliorated from 3.5 (2.5–4.5) to 3.0 (1.0‐4.0). Motor and sensory scores in OSIS score also improved. The median score of visual acuity remained stable until one patient withdrew with normal vision after the 4th visit. Hauser Ambulation Index decreased from 1.0 (0.0–3.0) to 0.0 (0.0–1.0).

### 3.3 Characteristics of immune indexes

**FIGURE 4:**
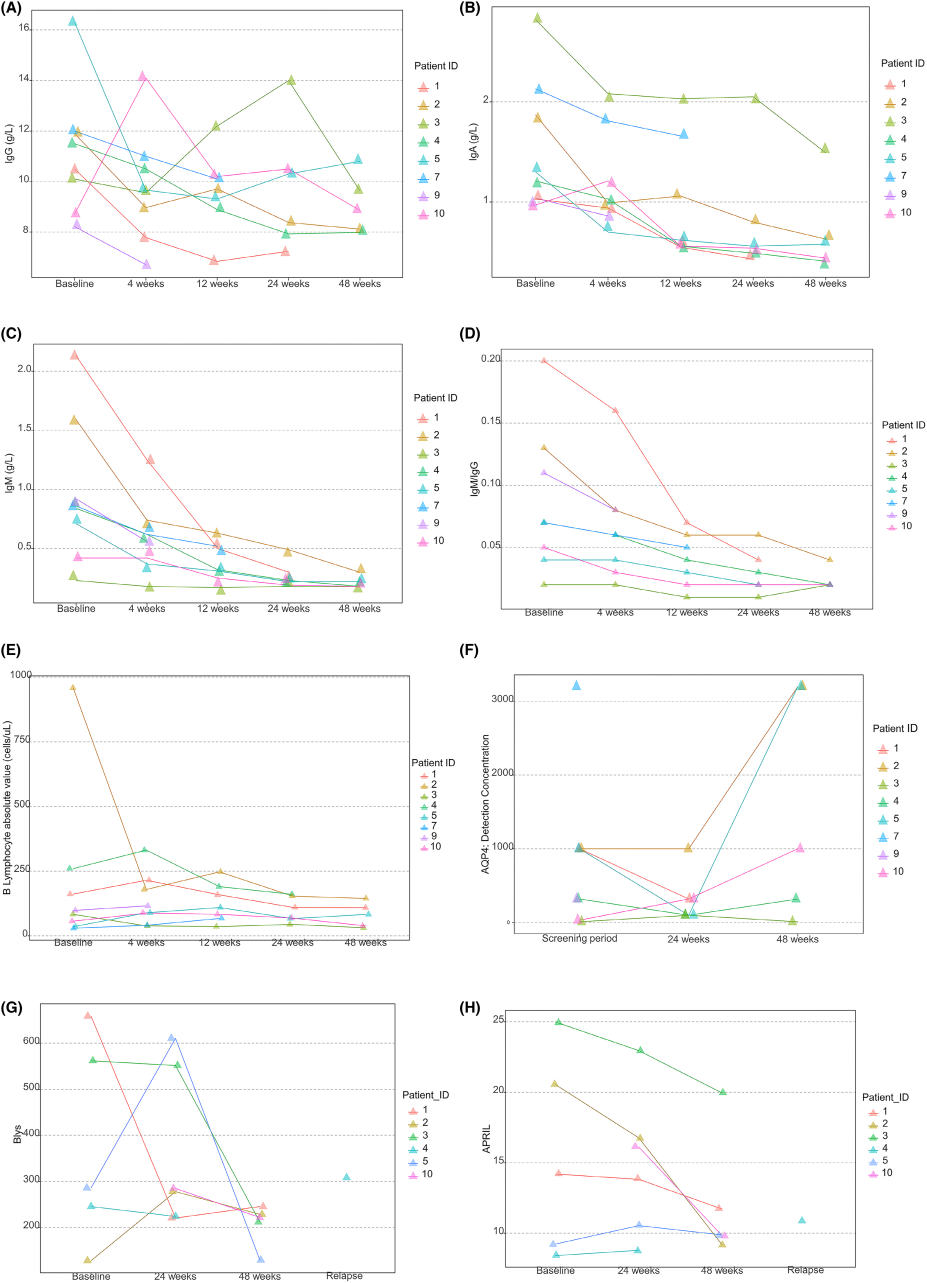
Immune analysis in serum. (A–D) Blood immunoglobulin analysis at baseline and week 4, 12, 24, 48. (E) CD19 + B lymphocyte count analysis by flow cytometry at baseline and week 4, 12, 24, 48. (F) Serum AQP4 antibody analysis by cell‐based transfection immunofluorescence assay (CBA) at baseline and week 24, 48. (G, H) BLys and APRIL analysis by ELISA at baseline and week 24, 48 or relapse

Both serum IgG and IgA decreased in 48 weeks, consistent with Telitacicept mechanism of action in depleting B lymphocytes. The serum IgM level decreased dramatically from 0.85 (0.23, 2.13) g/L at baseline to 0.18 (0.18, 0.3) g/L at 48th week. The IgM/IgG ratio was lower at 48th week of treatment and higher at the recurrence, compared to the baseline (Figure 4A–D). The CD19+ B lymphocyte count slowly increased to its peak value of 109.2 (35.4, 247.2) cells/μl at 12th week in relapse‐free patients from 90.5 (29.3, 958.8) cells/μl at baseline, but later gradually decreased to be 82.6 (30.7, 144.4) cells/μl at 48th week. The B lymphocyte count of the relapsed subjects significantly increased to 161 (29.3, 689.6) cells/μl (Figure 4E–F; Table S1). The serum AQP4 antibody titer tended to be stable. It increased during the last visit in some patients. The level of BLys and APRIL generally decreased along the course in relapse‐free participants, but increased at recurrence. Interestingly, Patient 5 had increased levels of both BLys and APRIL at 24 weeks, without recurrence during the study.

### 3.4 Characteristics of radiography

**FIGURE 5:**
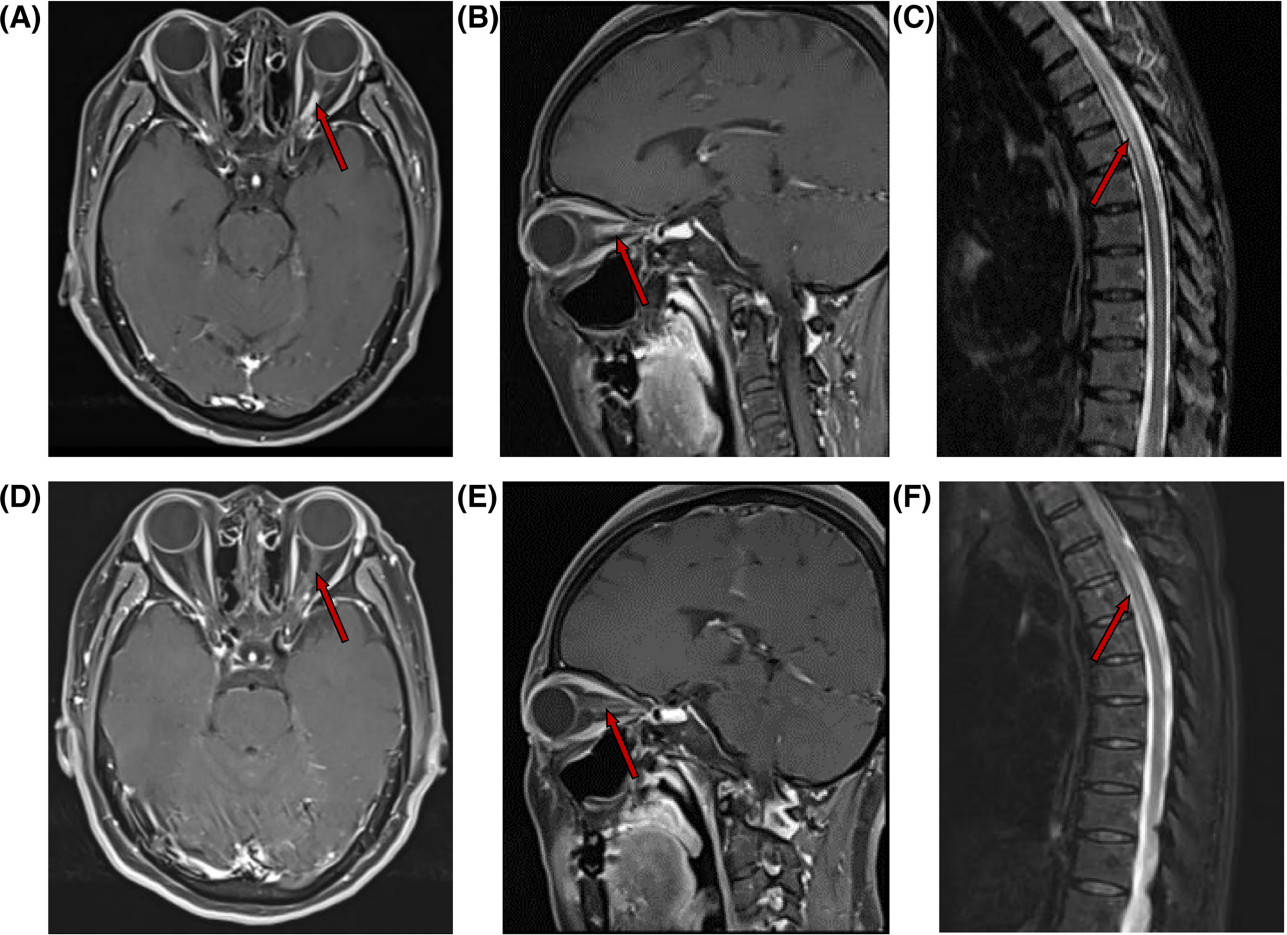
(A, B) Patient 2 hospitalized due to blindness of left eye for more than 1 week and narrowed vision of right eye for 4 days. Figure (A) shows enhanced signal of left optic nerve in gadolinium enhanced at baseline T1WI. Figure (B) shows gadolinium enhanced T1WI enhancement in sagittal position of left eye at baseline. (C) Thoracic MRI of Patient 1 at baseline. There was a high signal lesion of T2WI in the thoracic medulla at T3–T7 level. (D, E) Patient 2 was reexamined with MRI after 48 weeks of treatment. Enhanced signal of left optic nerve in gadolinium enhanced T1WI was improved. An orbital‐like enhancement was observed in the optic nerve sheath in gadolinium enhanced T1WI. The left optic nerve sheath was thickened, considering the chronic change of optic myelitis. (F) Thoracic MRI of Patient 1 at week 48. The high signal lesion of T2WI in the thoracic medulla at baseline diminished after Telitacicept treatment

Eight patients underwent MRI examination during the baseline period. Five patients without recurrence completed MRI examination at 48th week. No high‐signal intensities were observed in the brain scanning of the eight patients. Enhanced high‐signal lesions in the optic nerve were found in six patients at baseline. Thickening of optic nerve sheath and orbital enhancement were detected in five patients at 48th week. An increase in the total length of the optic nerve T2WI high‐signal intensities were observed on both sides. However, during this study, no aggravation in visual symptoms was likely due to adaptations in the chronic course of NMOSD (Figure 5). T1‐enhanced lesions in spinal cord resolved at 48th week. The number of T2WI high‐signal intensities also decreased from 1.5 (0, 3) at baseline to 1 (0, 2) at 48th week. The number of spinal cord segments involving high signal lesions on T2WI remained unchanged (Table S2).

### 3.5 Characteristics of OCT and VEP

The thickness of RNFL in OCT tended to be stable. In the left eye of patient 2, it gradually became thinner since the visit at 4th week. However, there was no significant change in the VEP examination and clinical symptoms.

The latency and amplitude of VEP P100 wave tended to be stable during the study. Due to the poor compliance of Patient 8, the P100 wave of VEP in binocular cortex was not recorded at 4th, 24th, 48th week (Table S2).

### 3.6 Safety assessment

A total of 43 adverse events were registered (AE). Injected site swelling was the most frequent adverse event, which was ameliorated within 2–4 months (Table 3). Patient 7 was affected by concomitant SLE and RA. She had a history of leukopenia and decreased neutrophil count and, during the study, her neutrophil count was less than 1.0 × 10^9^/L for two times. According to the protocol, the patient was removed from the study. The researchers considered that, for this patient, the decreased neutrophil and leukocyte count is likely attributable to either the administration of Telitacicept or concomitant SLE. Patient 3 and 4 were child‐bearing age females and they had amenorrhea and menorrhagia, respectively. Patient 3 had late menarche and irregular menstruation prior to this study. Patient 4 had similar symptoms before enrollment. Further investigation is required to determine whether these episodes were related to the intervention of this study.

**TABLE 3:** Summary of AEs and Patients with Reported AEs^a^ (*N* = 8)

Index	Patients with events, *n* (%)
Patients with at least 1 AE, *n* (%)	8 (100.0)
Patients with serious AEs, *n* (%)	0
Deaths, *n* (%)	0
Redness and swelling at injection site	4(50.0)
Abnormal in complete blood cell	3 (37.5)
Urinary tract infection	3 (37.5)
Menstrual disorder	2 (25.0)
paresthesia	3 (37.5)
Influenza	2 (25.0)
pulmonary nodule	2 (25.0)
Hyperlipidemia	2 (25.0)
Hematoma at venous catheterization	1 (12.5)
Oral ulcer	1 (12.5)
Fever	1 (12.5)
Conjunctivitis	1 (12.5)
Hypoglycemia	1 (12.5)
Hyperglycemia	1 (12.5)
Gastric distention	1 (12.5)
Anemia	1 (12.5)
Human papillomavirus infection, chronic cervicitis, mycoplasma infection	1 (12.5)
Sinus tachycardia	1 (12.5)
Fatigue	1 (12.5)
Articular pain	1 (12.5)
Constipation	1 (12.5)
Pulmonary exudation	1 (12.5)

Abbreviation: AE, Adverse Event.

^a^ A patient may be counted in more than 1 category and for more than 1 event (Medical Dictionary for Regulatory Activities [MedDRA 6.0]).

## 4 DISCUSSION

This is the first study to introduce Telitacicept following plasma exchange to recurrent NMOSD patients during acute phase. Plasma exchange removes pro‐inflammatory factors, providing a fresh start for Telitacicept to kick in. Telitacicept neutralizes the interaction between BLys and APRIL, resulting in inhibition of autoimmune humoral immunity and prolonged recurrent interval of NMOSD. In our study, the time of first recurrence was prolonged and the disability function score decreased, indicating a potiential effectiveness of Telitacicept in preventing the recurrence of NMOSD, avoiding the side effects of corticosteroid hormone. Adverse events were mild and resolved quickly, and no serious adverse event occurred during the study, indicating an acceptable safety profile of Telitacicept. The study showed that Telitacicept following plasma exchange may be a potential treatment option in patients with recurrent NMOSD.

Two out of eight patients relapsed with optic neuritis during the study. Patient 9 relapsed at the fourth (28 days) administration. This is likely due to the delayed effects of Telitacicept. Previous studies on RA and SLE showed that the serum level of TACI‐BLyS complex increased slowly and reached its peak in 5 to 29 days following Telitacicept administration.^28^ The CD19+ B lymphocyte count and the ratio of IgM/IgG, the levels of BLys and APRIL were also significantly increased at recurrence, suggesting the underlying dysregulated immune function. Two patients had both preceding fatigue due to either a long travel or sleep disruption before the onset of relapse. It is thus plausible that a delayed effect of Telitacicept, worsened by lack of sleep or rest, leads to the relapse of NMOSD. It remains to be determined whether Telitacicept provides limited benefits in optic neuropathy.

Most adverse events were self‐limited, and this result is similar to previous studies of Telitacicept in SLE and RA.^29^, ^30^ Patient 7 complicated with SLE and RA withdrew from the study because, for two times, the neutrophil count was less than 1.0 × 109/L. The patient had an history of low neutrophil count even less than 1.0 × 109/L before enrollment. Recurrent neutropenia is attributable to either Telitacicept or concomitant SLE.^31^ Therefore, complete blood cell count should be closely monitored especially in NMOSD patients with SLE. Additionally, two young women had menorrhagia or amenorrhea, both had a previous history of menstrual disorders. Thus, further evidence is needed to investigate whether Telitacicept affects the menstruation in women of childbearing age.

Previous studies show that AQP4 antibody titer has no positive correlation with the disease course.^32^ The antibody titer of AQP4 in serum tend to be stable during Telitacicept treatment. The potential mechanism needs further study. Despite a slow rise in the first 12 weeks, a significant decrease of CD19+ B lymphocyte count was observed at 48th week, consistent with previous studies of patients administered with Telitacicept in RA.^26^, ^28^ A notable decrease of serum IgM and IgM/IgG ratio were also detected at 48th week. Serum levels of Blys and APRIL decreased gradually after treatment, and increased at the time of recurrence, suggesting that Telitacicept inhibits B lymphocyte signals necessary for growth and activation. Telitacicept targeted to deprive part of B lymphocyte and reduced immunoglobulin production, thereby preventing diseases' recurrence.^27^ Therefore, Telitacicept might be safer than other B‐cell targeted drugs, such as anti‐CD20, because it might spare patients' prolonged B cell depletion.

This single‐center, single‐arm study had some limitations. As it was the first time to apply Telitacicept following plasma exchange to patients with NMOSD, only a small sample size of eight subjects in a single‐arm, uncontrolled study was approved by the Ethics Committee to reduce the potential risk and harms to patients. A large‐scale, randomized controlled trial is not possible without a preceding small‐scale early‐stage clinical study showing no potential safety concerns but with conceivable therapeutic effectiveness. In addition, long‐term studies beyond 48 weeks are needed. Although our work did not draw a definite conclusion concerning the safety and effectiveness, our work did provide important information of a novel alternative therapeutic strategy for NMOSD with probably better therapeutic potentials but with no serious safety issues observed so far, worthy of next stage clinical study.

## 5 CONCLUSION

In conclusion, this is the first study of Telitacicept following plasma exchange, a novel therapeutic strategy, in recurrent NMOSD. This exploratory study showed that Telitacicept following plasma exchange has the potential to be generally well tolerated in patients with NMOSD. This study may not have sufficient power to evaluate clinical efficacy. However, positive trends were seen for effects on recurrence of NMOSD. It may prolong the recurrence interval and reduce the annual count of recurrences of NMOSD. An additional investigation with a large‐scale, multicenter randomized controlled study is highly recommended.

## AUTHOR CONTRIBUTIONS

Conceptualization and methodology: Yangtai Guan, Ying Zhang and Yu Cai; Formal analysis and investigation: Jie Ding, Xianguo Jiang, Yong Hao. Shuting Pan, Meichun Gao, Nan Zhao, Ze Wang, Haojun Yu, Yuyan Jin, Huiying Qiu, Jiahui Xue, Quan Guo and Liping Ni; Writing – original draft preparation: Jie Ding; Writing – review and editing: Yangtai Guan, Yong Hao, Yu Cai and Xianguo Jiang; Funding acquisition: Yangtai Guan; Supervision: Ye Deng and Yan Lin.

## CONFLICT OF INTEREST

The authors declare that the research was conducted in the absence of any commercial or financial relationships that could be construed as a potential conflict of interest.

## INFORMED CONSENT

Written informed consent was obtained from participants or their guardians.

## Supporting information


Tables S1‐S2

## Data Availability

The data that support the findings of this study are available from the corresponding author upon reasonable request.
