# Supplementary material for: Telitacicept following plasma exchange in the treatment of subjects with recurrent neuromyelitis optica spectrum disorders: A single‐center, single‐arm, open‐label study
Source: CNS Neurosci Ther. 2022 Jul 18;28(10):1613–23. doi: 10.1111/cns.13904 (PMC9437241; doi:10.1111/cns.13904)
Supplement: Supplementary file 1 — Tables S1‐S2 [file CNS-28-1613-s001.docx]

**Table S1. Characteristics of immune indexes** **before and after treatment**

| **Index** | Baseline(n=8) | 4^th^ week  (n=8) | 12^th^ week  (n=7) | 24^th^ week  (n=6) | 48^th^ week  (n=5) | recurrence(n=2) |
| --- | --- | --- | --- | --- | --- | --- |
| AQP4 (positive), n(%) | 8(100) | / | / | 6(100) | 5(100) | / |
| IgG (g/L) | 11.00(8.22, 16.3) | 9.61(6.71, 14.10) | 9.71(6.84, 12.20) | 9.34(7.24, 14.00) | 8.86(7.99, 10.80) | 6.50(6.33, 6.66) |
| IgA (g/L) | 1.26(0.97, 2.82) | 1.01(0.7, 2.08) | 0.62(0.55, 2.03) | 0.55(0.43, 2.05) | 0.58(0.41, 1.49) | 0.525(0.39, 0.66) |
| IgM (g/L)  *values<0.18werereplacedby0.18or0.17 | 0.85(0.23, 2.13) | 0.59(0.18, 1.25) | 0.32(0.17, 0.63) | 0.225(0.18, 0.49) | 0.18(0.18, 0.3) | 0.305(0.2, 0.41) |
| IgM/IgG | 0.072(0.023, 0.203) | 0.058(0.019, 0.161) | 0.036(0.014, 0.073) | 0.025(0.013, 0.058) | 0.02(0.019, 0.037) | 0.047(0.032, 0.062) |
| C3 (g/L) | 0.96(0.79, 1.26) | 1.025(0.61, 1.44) | 1.02(0.62, 1.42) | 1.065(0.83, 1.37) | 1.1(0.82, 1.39) | 1.005(0.94, 1.07) |
| C4(g/L) | 0.18(0.05, 0.26) | 0.22(0.15, 0.3) | 0.24(0.13, 0.38) | 0.26(0.2, 0.31) | 0.24(0.21, 0.25) | 0.155(0.11, 0.2) |
| Lymphocyte count (10*9/L) | 1.06(0.24, 2.55) | 2.09(1.29, 6.67) | 2.18(0.77, 3.44) | 1.75(0.93, 1.89) | 1.58(0.64, 3.19) | 1.395(0.91, 1.88) |
| B Lymphocyte count (cells/uL) | 90.5(29.3, 958.8) | 102.05(38.2, 331.1) | 109.2(35.4, 247.2) | 89.05(43.9, 160.5) | 82.6(30.7, 144.4) | 161(29.3, 689.6) |
| T Lymphocyte count(cells/uL) | 950.2(182.9, 1297.9) | 1507.95(526, 2257.5) | 1258.6(627.5, 2192.2) | 1112.7(748, 1480.2) | 1240.3(52.5, 1859.8) | 1182.95(799.9, 1566) |
| Th Lymphocyte count(cells/uL) | 376.3(109.2, 958.8) | 707.8(182.9, 1246.9) | 612.6(157.9, 980.4) | 665(193.2, 721.4) | 745.8(154.2, 979.3) | 491.95(263.9, 720) |
| Ts Lymphocyte count(cells/uL) | 327.15(58.8, 657.5) | 587(83.69, 1033.5) | 589.1(295, 1234) | 417(242.8, 801) | 365(249.6, 925.3) | 564(483.2, 644.8) |
| NK Lymphocyte count(cells/uL) | 83(7.7, 846) | 221.7(32.6, 1185.2) | 344(20.2, 1090.5) | 275.95(95.9, 693.6) | 282.8(92.8, 1196.3) | 96.35(59.2, 133.5) |

AQP4, aquaporin 4; IgG, Immunoglobulin G; IgA, Immunoglobulin A; IgM, Immunoglobulin M; C3, complement 3; C4, complement 4; NK, natural killer.

**Table S2. Clinical, electrophysiological, and imaging characteristics of patients before and after treatment**

| **Index** | Baseline(n=8) | 4^th^ week  (n=8) | 12^th^ week  (n=7) | 24^th^ week  (n=6) | 48^th^ week  (n=5) | Recurrence1 | Recurrence2 |
| --- | --- | --- | --- | --- | --- | --- | --- |
| Expanded Disability Status Scale (EDSS), median (range) | 3.5(2.5-4.5) | 2.3(1-4.5) | 2.5(1-4.5) | 2.5(1-4.5) | 3(1-4) | 3 | 4 |
| Optic spinal Impairment Score (OSIS), median (range) |  | | | | | | |
| Visual Acuity | 6.0(2.0-8.0) | 2.5(0-8) | 0(0-8) | 3(0-8) | 6(0-8) | 6 | 6 |
| Motor | 1.0(0.0-3.0) | 1(0-3) | 1(0-1) | 1(0-1) | 0.5(0-1) | 0 | 1 |
| Sensory | 2.0(0.0-3.0) | 0(0-2) | 0(0-2) | 0(0-2) | 0(0-2) | 0 | 0 |
| Sphincter | 0.0(0.0-1.0) | 0(0-0) | 0(0-0) | 0(0-0) | 0(0-0) | 0 | 0 |
| Hauser Ambulation Index median (range) | 1.0(0.0-3.0) | 0.0(0.0-1.0) | 0.0(0.0-1.0) | 0.0(0.0-1.0) | 0.0(0.0-1.0) | 0 | 0 |
| Retinal Nerve Fiber Layer Thickness (OCT) |  | | | | | | |
| Left, median (range) | 92.0(55.0-108.0)(n=7) | 87.0(60.0-109.0)(n=7) | 80.0(57.0-109.0)(n=5) | 63.0(54.0-104.0)(n=4) | 58.5(43.0-102.0)(n=4) | 95 | 60 |
| Abnormal, n (%) | 3(42.9) | 4(57.1) | 2(40.0) | 3(75.0) | 3(60.0) | No | Yes |
| Right, median (range) | 74.0(49.0-102.0) (n=7) | 87.0(59.0-105.0) (n=7) | 74.5(52.0-104.0) (n=5) | 59.0(46.0-98.0) (n=4) | 54.5(47.0, 98.0) (n=4) | 89 | 72 |
| Abnormal, n (%) | 4(57.1)(n=7) | 4(57.1) | 3(60.0) | 3(75.0) | 3(60.0) | No | Yes |
| Visual Evoked Potential |  | | | | | | |
| P100 Latency |  | | | | | | |
| Left, median (range) | 108.0(13.0-125.0) | 97.2(81-118) | 104(95-117) | 111(95-115) | 107(96.3-113) | 133 | NA |
| NP, n (%) | 1(12.5) | 2(25.0) | 1(14.3) | 2(33.3) | 2(40.0) |  |  |
| Right- median (range) | 105.0(100.0-130.0) | 100(93.8-120) | 114(96-162) | 107(99.3-142) | 122(103-140) | 98.2 | 122 |
| NP, n (%) | 5(62.5) | 3(37.5) | 2(28.6) | 3(50.0) | 3(60.0) |  |  |
| P100 Amplitude |  | | | | | | |
| Left, median (range) | 3.7(2.3-9.4) | 5.4(2.10-8.7) | 5.5(2.4-7.4) | 5.9(4.5-8.9) | 6.6(6.5-11.3) | 1.46 | NA |
| NP, n (%) | 1(12.5) | 2(25.0) | 1(14.3) | 2(33.3) | 2(40.0) |  |  |
| Right, median (range) | 5.3(2.3-16.5) | 4.0(2.8-13.3) | 5.1(2.7-17.1) | 6.0(3.2-16.7) | 10.2(3.7-16.7) | 6.5 | 6.2 |
| NP, n (%) | 4(50.0) | 3(37.5) | 2(28.6) | 3(50.0) | 3(60.0) |  |  |
| Abnormal, n (%) | 7(87.5) | 6(75.0) | 6(85.7) | 6(100) | 5(100) | YES | YES |
| MRI |  | | | | | | |
| Enhanced high-signal lesions in the optic nerve, n (%) | 6(75.0) | / | / | / | 5(100) | 1(100) | 1(100) |
| length of T2WI high-signal intensities of the optic nerve |  | | | | | | |
| Left, median (range) | 36.9(0, 87.5) | / | / | / | 80.7(0, 88.1) | 34.8 | 82.8 |
| Right, median (range) | 21.1(0, 80.8) | / | / | / | 32.3(0, 75) | 27.0 | 62.1 |
| T1-enhanced lesions in spinal cord, median (range) | 1(0, 4) | / | / | / | 0(0, 1) | 0 | 0 |
| number of T2WI high-signal intensities in spinal cord, median (range) | 1.5(0-3) | / | / | / | 1(0-2) | 2 | 1 |
| number of spinal segments with T2WI high signal, median (range) | 5(0, 5.5) | / | / | / | 5(0, 5) | 5 | 2 |

NP, no potential；MRI, magnetic resonance imaging；T2WI, T2 weighted imaging
